# Complete genome sequence of the moderately thermophilic mineral-sulfide-oxidizing firmicute *Sulfobacillus acidophilus* type strain (NAL^T^)

**DOI:** 10.4056/sigs.2736042

**Published:** 2012-07-20

**Authors:** Iain Anderson, Olga Chertkov, Amy Chen, Elizabeth Saunders, Alla Lapidus, Matt Nolan, Susan Lucas, Nancy Hammon, Shweta Deshpande, Jan-Fang Cheng, Cliff Han, Roxanne Tapia, Lynne A. Goodwin, Sam Pitluck, Konstantinos Liolios, Ioanna Pagani, Natalia Ivanova, Natalia Mikhailova, Amrita Pati, Krishna Palaniappan, Miriam Land, Chongle Pan, Manfred Rohde, Rüdiger Pukall, Markus Göker, John C. Detter, Tanja Woyke, James Bristow, Jonathan A. Eisen, Victor Markowitz, Philip Hugenholtz, Nikos C. Kyrpides, Hans-Peter Klenk, Konstantinos Mavromatis

**Affiliations:** 1 DOE Joint Genome Institute, Walnut Creek, California, USA; 2 Los Alamos National Laboratory, Bioscience Division, Los Alamos, New Mexico, USA; 3 Biological Data Management and Technology Center, Lawrence Berkeley National Laboratory, Berkeley, California, USA; 4 Oak Ridge National Laboratory, Oak Ridge, Tennessee, USA; 5 HZI – Helmholtz Centre for Infection Research, Braunschweig, Germany; 6 Leibniz Institute DSMZ - German Collection of Microorganisms and Cell Cultures, Braunschweig, Germany; 7 University of California Davis Genome Center, Davis, California, USA; 8 Australian Centre for Ecogenomics, School of Chemistry and Molecular Biosciences, The University of Queensland, Brisbane, Australia

**Keywords:** aerobic, motile, Gram-positive, acidophilic, moderately thermophilic, sulfide- and iron-oxidizing, biomining, autotrophic, mixotrophic, soil, *insertis sedis*, *Clostridiales*, GEBA

## Abstract

*Sulfobacillus acidophilus* Norris *et al.* 1996 is a member of the genus *Sulfobacillus* which comprises five species of the order *Clostridiales*. *Sulfobacillus* species are of interest for comparison to other sulfur and iron oxidizers and also have biomining applications. This is the first completed genome sequence of a type strain of the genus *Sulfobacillus*, and the second published genome of a member of the species *S. acidophilus*. The genome, which consists of one chromosome and one plasmid with a total size of 3,557,831 bp harbors 3,626 protein-coding and 69 RNA genes, and is a part of the ***G****enomic* ***E****ncyclopedia of* ***Bacteria**** and* ***Archaea***** project.

## Introduction

The genus *Sulfobacillus* currently consists of five species [1], all of which are mildly thermophilic or thermotolerant acidophiles [2]. Sulfobacilli grow mixotrophically by oxidizing ferrous iron, sulfur, and mineral sulfides in the presence of yeast extract or other organic compounds [3]. Some can also grow autotrophically [2,3]. The strains that have been tested are capable of anaerobic growth using Fe^+3^ as an electron acceptor [2,4]. The genus *Sulfobacillus*, along with the genus *Thermaerobacter*, have only tentatively been assigned to a family, “*Clostridiales* Family XVII *incertae sedis”*. This group may form a deep branch within the phylum *Firmicutes* or may constitute a new phylum [5]. Strain NAL^T^ (= DSM 10332 = ATCC 700253) is the type strain of the species *Sulfobacillus acidophilus*. The genus name was derived from the Latin words 'sulfur' and 'bacillus' meaning 'small sulfur-oxidizing rod' [6]. The species epithet is derived from the Neo-Latin words 'acidum', acid, and 'philus', loving, meaning acid-loving [3]. The first genome from a member of the species *S. acidophilus*, strain TPY, which was isolated from a hydrothermal vent in the Pacific Ocean, was recently sequenced by Li *et al.* [7]. Here we present a summary classification and a set of features for *S. acidophilum* strain NAL^T^, together with the description of the complete genomic sequencing and annotation.

## Classification and features

A representative genomic 16S rRNA sequence of *S. acidophilus* NAL^T^ was compared using NCBI BLAST [8,9] under default settings (e.g., considering only the high-scoring segment pairs (HSPs) from the best 250 hits) with the most recent release of the Greengenes database [10] and the relative frequencies of taxa and keywords (reduced to their stem [11]) were determined, weighted by BLAST scores. The most frequently occurring genera were *Sulfobacillus* (81.9%), *Thermaerobacter* (8.0%), *Laceyella* (2.8%), 'Gloeobacter' (2.1%) and 'Synechococcus' (2.0%) (76 hits in total). Regarding the six hits to sequences from members of the species, the average identity within HSPs was 98.9%, whereas the average coverage by HSPs was 97.2%. Regarding the 23 hits to sequences from other members of the genus, the average identity within HSPs was 93.1%, whereas the average coverage by HSPs was 81.2%. Among all other species, the one yielding the highest score was “*Sulfobacillus yellowstonensis*” (AY007665), which corresponded to an identity of 99.4% and an HSP coverage of 97.0%. (Note that the Greengenes database uses the INSDC (= EMBL/NCBI/DDBJ) annotation, which is not an authoritative source for nomenclature or classification.) The highest-scoring environmental sequence was HQ730681 ('Microbial Anaerobic Sediments Tinto River: Natural Acid and Heavy Metals Content extreme acid clone SN1 2009 12D'), which showed an identity of 94.5% and an HSP coverage of 99.0%. The most frequently occurring keywords within the labels of all environmental samples which yielded hits were 'acid' (4.8%), 'soil' (4.5%), 'hydrotherm' (3.7%), 'microbi' (3.7%) and 'mine' (3.0%) (172 hits in total). These keywords correspond well to the environment from which strain NAL^T^ was isolated. Environmental samples that yielded hits of a higher score than the highest scoring species were not found.

**Figure 1:**
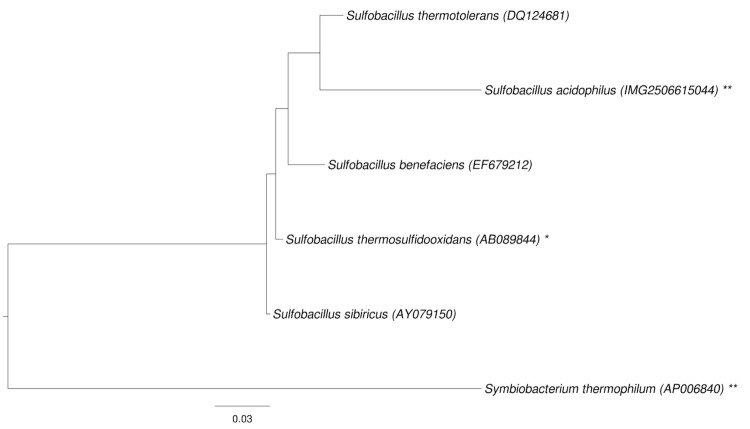
Phylogenetic tree highlighting the position of *S. acidophilus* relative to the type strains of the other species within the genus *Sulfobacillus*. The tree was inferred from 1,422 aligned characters [12,13] of the 16S rRNA gene sequence under the maximum likelihood (ML) criterion [14]. The comparatively closely related genus *Symbiobacterium* [15] was included for rooting the tree. The branches are scaled in terms of the expected number of substitutions per site. Numbers adjacent to the branches, if any, are support values from 1,000 ML bootstrap replicates [16] (left) and from 1,000 maximum parsimony bootstrap replicates [17] (right) if larger than 60% (i.e., there were none). Lineages with type strain genome sequencing projects registered in GOLD [18] are labeled with one asterisk, those also listed as 'Complete and Published' with two asterisks [19].

Figure 1 shows the phylogenetic neighborhood of *S. acidophilus* NAL^T^ in a 16S rRNA based tree. The sequences of the five 16S rRNA gene copies in the genome differ from each other by up to eight nucleotides, and differ by up to four nucleotides from the previously published 16S rRNA sequence (AB089842), which contains two ambiguous base calls.

**Figure 2:**
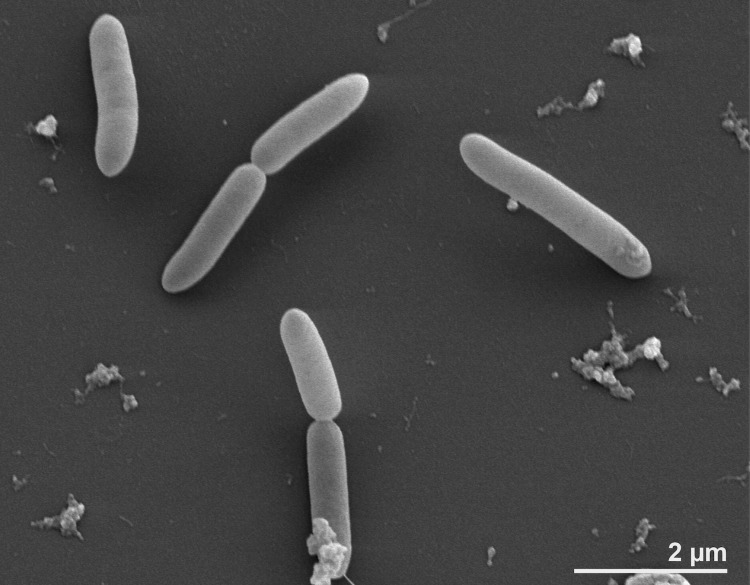
Scanning electron micrograph of *S. acidophilus* NAL^T^

Cells of *S. acidophilus* NAL^T^ are rods 3.0-5.0 μm in length and 0.5-0.8 μm in width (Table 1 and Figure 2) [3]. Cells are Gram-positive and form spherical endospores [3]. Flagella were not observed [3]. Strain NAL^T^ was found to grow between 28°C and 62°C with an optimum at 48°C [35]. The upper and lower temperatures for growth were not determined but were predicted to be 10°C and 62°C [35]. The pH range for growth was 1.6-2.3 with an optimum at 1.8 [35]. Three strains of *S. acidophilus* have been found to be facultative anaerobes that are able to use Fe^+3^ as an electron acceptor under anaerobic conditions [4]; but strain NAL^T^ was not tested in this study. Strain NAL^T^ can grow autotrophically or mixotrophically by oxidizing Fe^+2^, sulfur, or mineral sulfides or heterotrophically on yeast extract [3]. *S. acidophilus* and other sulfobacilli have potential applications in biomining. Strain NAL^T^ increased the leaching of numerous mineral sulfides [35], however, its sensitivity to low concentrations of metals may limit its usefulness in biomining [35].

**Table 1:** Classification and general features of *S. acidophilus* NAL^T^ according to the MIGS recommendations [20] and the NamesforLife database [21].

MIGS ID	Property	Term	Evidence code
	Current classification	Domain *Bacteria*	TAS [22]
		Phylum “*Firmicutes*”	TAS [23-25]
		Class *Clostridia*	TAS [26,27]
		Order *Clostridiales*	TAS [28,29]
		Family “XVII *incertae sedis*”	TAS [5,30]
		Genus *Sulfobacillus*	TAS [31-33]
		Species *Sulfobacillus acidophilus*	TAS [3,34]
		Type strain NAL	TAS [3]
	Gram stain	positive	TAS [3]
	Cell shape	rods	TAS [3]
	Motility	non-motile	NAS
	Sporulation	spherical endospores	TAS [3]
	Temperature range	not reported	
	Optimum temperature	48°C	TAS [35]
	Salinity	not reported	
MIGS-22	Oxygen requirement	facultative anaerobe	TAS [4]
	Carbon source	CO_2_, organic compounds	TAS [3]
	Energy metabolism	autotrophic, mixotrophic, heterotrophic	TAS [3]
MIGS-6	Habitat	acidic sulfidic and sulfurous sites	TAS [35]
MIGS-15	Biotic relationship	free-living	TAS [3]
MIGS-14	Pathogenicity	none	NAS
	Biosafety level	1	TAS [36]
	Isolation	coal spoil heap	TAS [3]
MIGS-4	Geographic location	Alvecote, North Warwickshire, UK	TAS [3]
MIGS-5	Sample collection time	1988	TAS [3]
MIGS-4.1	Latitude	52.638	TAS [3]
MIGS-4.2	Longitude	-1.641	TAS [3]
MIGS-4.3	Depth	not reported	
MIGS-4.4	Altitude	not reported	

Evidence codes - IDA: Inferred from Direct Assay (first time in publication); TAS: Traceable Author Statement (i.e., a direct report exists in the literature); NAS: Non-traceable Author Statement (i.e., not directly observed for the living, isolated sample, but based on a generally accepted property for the species, or anecdotal evidence). These evidence codes are from the Gene Ontology project [37]. If the evidence code is IDA, then the property was directly observed for a living isolate by one of the authors or an expert mentioned in the acknowledgements.

## Genome sequencing and annotation

### Genome project history

This organism was selected for sequencing on the basis of its phylogenetic position [38], and is part of the ***G****enomic* ***E****ncyclopedia of* ***Bacteria**** and* ***Archaea***** project [39]. The genome project is deposited in the Genomes OnLine Database [18] and the complete genome sequence is deposited in GenBank. Sequencing, finishing and annotation were performed by the DOE Joint Genome Institute (JGI). A summary of the project information is shown in Table 2.

**Table 2:** Genome sequencing project information

**MIGS ID**	**Property**	**Term**
MIGS-31	Finishing quality	Finished
MIGS-28	Libraries used	Four genomic libraries: one 454 pyrosequence standard library, two 454 PE libraries (6 kb and 10 kb insert size), one Illumina library
MIGS-29	Sequencing platforms	Illumina GAii, 454 GS FLX Titanium
MIGS-31.2	Sequencing coverage	168.4 × Illumina; 51.2 × pyrosequence
MIGS-30	Assemblers	Newbler version 2.3-PreRelease-6/30/2009, Velvet 1.0.13, phrap version SPS - 4.24
MIGS-32	Gene calling method	Prodigal 1.4, GenePRIMP
	INSDC ID	CP003179 (chromosome) CP003180 (plasmid, unnamed)
	Genbank Date of Release	December 14, 2011
	GOLD ID	Gc02053
	NCBI project ID	40777
	Database: IMG-GEBA	2506520015
MIGS-13	Source material identifier	DSM 10332
	Project relevance	Tree of Life, GEBA, biomining

### Growth conditions and DNA isolation

*S. acidophilus* strain NAL^T^, DSM 10332, was grown in DSMZ medium 709 (*Acidomicrobium* medium) [40] at 45°C. DNA was isolated from 0.5-1 g of cell paste using MasterPure Gram-positive DNA purification kit (Epicentre MGP04100) following the standard protocol as recommended by the manufacturer with modification st/LALM for cell lysis as described in Wu *et al*. 2009 [39]. DNA is available through the DNA Bank Network [41].

### Genome sequencing and assembly

The genome was sequenced using a combination of Illumina and 454 sequencing platforms. All general aspects of library construction and sequencing can be found at the JGI website [42]. Pyrosequencing reads were assembled using the Newbler assembler (Roche). The initial Newbler assembly consisting of 104 contigs in three scaffolds was converted into a phrap [43] assembly by making fake reads from the consensus, to collect the read pairs in the 454 paired end library. Illumina GAii sequencing data (599.7 Mb) were assembled with Velvet [44] and the consensus sequences were shredded into 1.5 kb overlapped fake reads and assembled together with the 454 data. The 454 draft assembly was based on 143.7 Mb of 454 draft data and all of the 454 paired-end data. Newbler parameters were -consed -a 50 -l 350 -g -m -ml 20. The Phred/Phrap/Consed software package [43] was used for sequence assembly and quality assessment in the subsequent finishing process. After the shotgun stage, reads were assembled with parallel phrap (High Performance Software, LLC). Possible mis-assemblies were corrected with gapResolution (C. Han, unpublished), Dupfinisher [45], or sequencing cloned bridging PCR fragments with subcloning. Gaps between contigs were closed by editing in Consed, PCR and Bubble PCR primer walks (J.-F. Chang, unpublished). A total of 640 additional reactions and eight shatter libraries were necessary to close gaps and to raise the quality of the finished sequence. Illumina reads were also used to correct potential base errors and increase consensus quality using the software Polisher developed at JGI [46]. The error rate of the completed genome sequence is less than 1 in 100,000. Together, the combination of the Illumina and 454 sequencing platforms provided 219.6 × coverage of the genome. The final assembly contained 612,059 pyrosequence and 16,626,072 Illumina reads.

### Genome annotation

Genes were identified using Prodigal [47] as part of the Oak Ridge National Laboratory genome annotation pipeline, followed by a round of manual curation using the JGI GenePRIMP pipeline [48]. The predicted CDSs were translated and used to search the National Center for Biotechnology Information (NCBI) nonredundant database, UniProt, TIGR-Fam, Pfam, PRIAM, KEGG, COG, and InterPro databases. Additional gene prediction analysis and functional annotation was performed within the Integrated Microbial Genomes - Expert Review (IMG-ER) platform [49].

## Genome properties

**Figure 3:**
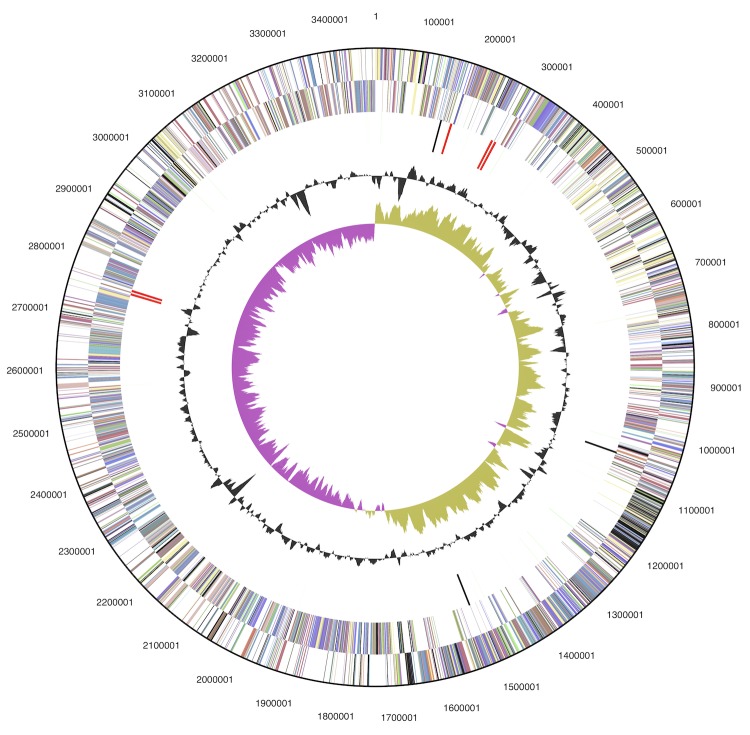
Graphical map of the chromosome. From outside to the center: Genes on forward strand (colored by COG categories), Genes on reverse strand (colored by COG categories), RNA genes (tRNAs green, rRNAs red, other RNAs black), GC content, GC skew.

**Figure 4:**
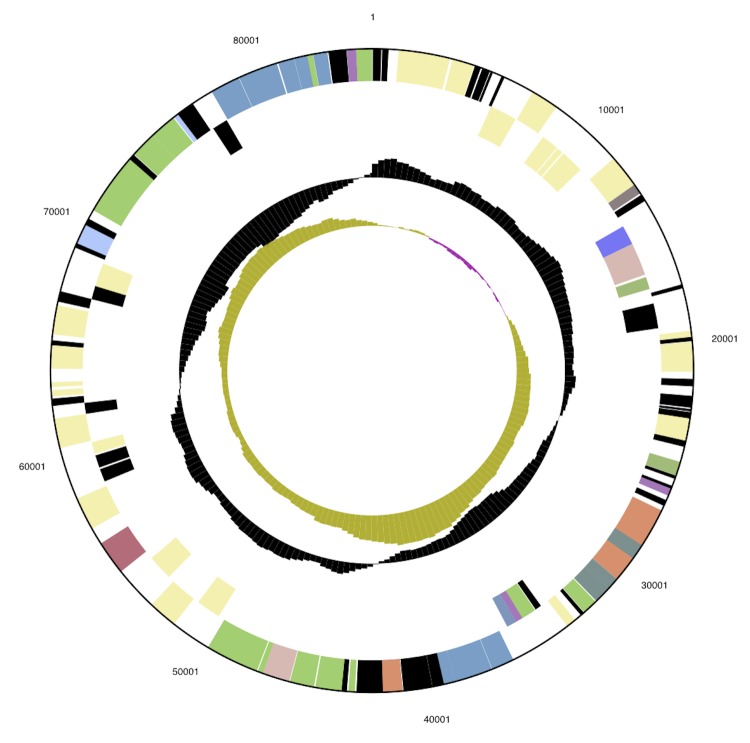
Graphical map of the plasmid. From outside to the center: Genes on forward strand (colored by COG categories), Genes on reverse strand (colored by COG categories), RNA genes (tRNAs green, rRNAs red, other RNAs black), GC content, GC skew.

The genome consists of one circular chromosome of 3,472,898 bp and one circular plasmid of 84,933 bp length with an overall G+C content of 56.8% (Table 3 and Figures 3 and 4). Based on coverage of 454 paired ends, the plasmid may be inserted into the chromosome in about half of the population. Of the 3,695 genes predicted, 3,626 are protein-coding genes, and 69 are RNAs; 155 pseudogenes were also identified. The majority of the protein-coding genes (68.3%) were assigned a putative function while the remaining ones were annotated as hypothetical proteins. The distribution of genes into COGs functional categories is presented in Table 4.

**Table 3:** Genome Statistics

**Attribute**	**Value**	**% of Total^a^**
Genome size (bp)	3,557,831	100.00%
DNA coding region (bp)	3,106,298	87.31%
DNA G+C content (bp)	2,019,235	56.75%
Number of replicons	2	
Extrachromosomal elements	1	
Total genes	3,695	
RNA genes	69	
rRNA operons	5	
Protein-coding genes	3,626	100.00%
Pseudo genes	155	4.27%
Genes with function prediction	2,475	68.26%
Genes in paralog clusters	1,896	52.29%
Genes assigned to COGs	2,740	75.57%
Genes assigned Pfam domains	413	11.39%
Genes with signal peptides	652	17.98%
Genes with transmembrane helices	910	25.10%
CRISPR repeats	2	

a) The total is based on either the size of the genome in base pairs or the total number of protein coding genes in the annotated genome.

**Table 4:** Number of genes associated with the general COG functional categories

**Code**	**value**	**%age^a^**	**Description**
J	149	4.1	Translation, ribosomal structure and biogenesis
A	0	0.0	RNA processing and modification
K	188	5.2	Transcription
L	269	7.4	Replication, recombination and repair
B	1	0.0	Chromatin structure and dynamics
D	26	0.7	Cell cycle control, cell division, chromosome partitioning
Y	0	0.0	Nuclear structure
V	34	0.9	Defense mechanisms
T	111	3.1	Signal transduction mechanisms
M	149	4.1	Cell wall/membrane/envelope biogenesis
N	47	1.3	Cell motility
Z	0	0.0	Cytoskeleton
W	0	0.0	Extracellular structures
U	62	1.7	Intracellular trafficking, secretion, and vesicular transport
O	129	3.6	Posttranslational modification, protein turnover, chaperones
C	244	6.7	Energy production and conversion
G	215	5.9	Carbohydrate transport and metabolism
E	257	7.1	Amino acid transport and metabolism
F	89	2.5	Nucleotide transport and metabolism
H	153	4.2	Coenzyme transport and metabolism
I	130	3.6	Lipid transport and metabolism
P	121	3.3	Inorganic ion transport and metabolism
Q	81	2.2	Secondary metabolites biosynthesis, transport and catabolism
R	326	9.0	General function prediction only
S	239	6.6	Function unknown
-	886	24.4	Not in COGs

a) The percentage is based on the total number of protein coding genes in the annotated genome.

## Insights into the genome sequence

### Comparative genomics

While the sequencing of the genome described in this paper was underway, Li *et al.* from the Third Institute of Oceanography, Xiamen, China published the complete genome sequence of strain TPY [7]. The two genomes differ in size by less than 7,000 bp. Here, we take the opportunity to compare the completed genome sequences from these two stains, NAL^T^ and TPY, both belonging to *S. acidophilus*. While the biological material for the type stain, NAL^T^, is publicly available from the DSMZ open collection for postgenomic analyses, no source of the biological material (MIGS-13 criterion, see Table 2) of strain TPY was provided by Li *et al.* [7].

To estimate the overall similarity between the genomes of strains NAL^T^ and TPY (Genbank accession number: CP002901), the GGDC-Genome-to-Genome Distance Calculator [50,51] was used. The system calculates the distances by comparing the genomes to obtain HSPs (high-scoring segment pairs) and interfering distances from three formulae (HSP length / total length; identities / HSP length; identities / total length). The comparison of the genomes of strains NAL^T^ and TPY revealed that 99.65% of the average of the genome lengths are covered with HSPs. The identity within these HSPs was 99.01%, whereas the identity over the whole genome (counting regions not covered by HSPs as non-identical) was 98.67%. The inferred digital DNA-DNA hybridization values for the two strains are 96.47% (formula 1 in [51]), 86.08% (formula 2 in [51]) and 97.05% (formula 3 in [51]), respectively. These results clearly demonstrate that according to the whole genome sequences of strains NAL^T^ and TPY, the similarity is very high, supporting the membership of both strains in the same species.

The comparison of the number of genes belonging to the different COG categories revealed few differences between the genomes of strains NAL^T^ and TPY. Strain NAL^T^ has 2,740 genes with COGs assigned, while strain TPY has 2,700. We analyzed the differences in COG assignment between the two strains and found that in almost all cases they could be explained by differences in the gene calls or pseudogene assignment, i.e. in one genome two parts of a pseudogene were called as two separate genes, while in the other genome they were combined into one pseudogene. The only clear case of a difference in gene content between the two strains is the presence of a transposable element consisting of two genes (Sulac_1668, Sulac_1669) disrupting a subunit of a potassium transporter (Sulac_1667) in strain NAL^T^. There were also cases where a gene in one strain was split into two genes in the other strain. For example, Sulac_2178 corresponds to TPY_1983 and TPY1984, and Sulac_0347 corresponds to TPY_0381 and TPY_0382. In both cases the differences are due to a single base indel.

A dot plot showed that there are large blocks of synteny between the two genomes with some rearrangements (data not shown). The genes found on the plasmid in strain NAL^T^ are found in two regions of the chromosome in strain TPY. Sulac_3528-3555 corresponds to TPY_0524-0552, while Sulac_3556-3626 corresponds to TPY_2310-2244. This suggests that in strain TPY, the plasmid was inserted into the chromosome and then split into two pieces.

We analyzed CRISPR repeats with the CRISPR Recognition Tool [52] and found major differences between the two strains. They both have two regions of CRISPR repeats, but the strain TPY repeat regions have 8 and 9 repeats while the strain NAL^T^ repeat regions have 27 and 43 repeats. All of the spacers in the TPY repeat regions are found in NAL^T^, but NAL^T^ has many additional spacers. This agrees with previous results suggesting that CRISPRs evolve quickly, and differences can be found in closely related strains [53].
